# Supplementary material for: The Global Trend of Microplastic Research in Freshwater Ecosystems
Source: Toxics. 2023 Jun 17;11(6):539. doi: 10.3390/toxics11060539 (PMC10303708; doi:10.3390/toxics11060539)
Supplement: Supplementary file 1 [file toxics-11-00539-s001.zip › toxics-2387221-supplementary.pdf]

Supporting information

The Global Trend of Microplastic Research in Freshwater Ecosystems

Yaochun Wang[1], Guohao Liu[1], Yixia Wang[1], Hongli Mu[1], Xiaoli Shi[1], Chao Wang[2], Naicheng Wu[1]*

[1] Department of Geography and Spatial Information Techniques, Ningbo University, Ningbo 315211, China

[2] Pearl River Fisheries Research Institute, Chinese Academy of Fishery Sciences, Guangzhou 510380, China

* Corresponding author: Naicheng Wu (naichengwu88@gmail.com)

Table S1. Countries involved in microplastics-related research and the volume of their publications, 2013-2017

From	To	Frequency
ARGENTINA	URUGUAY	2
AUSTRALIA	BANGLADESH	1
AUSTRALIA	BRUNEI	1
AUSTRALIA	EGYPT	1
AUSTRALIA	GHANA	1
AUSTRALIA	IRAN	1
AUSTRALIA	LITHUANIA	1
AUSTRALIA	MALAYSIA	1
AUSTRALIA	NORWAY	1
AUSTRALIA	PAKISTAN	1
AUSTRALIA	POLAND	1
AUSTRALIA	RUSSIA	2
AUSTRALIA	SAUDI ARABIA	1
AUSTRALIA	THAILAND	1
AUSTRALIA	VIETNAM	1
BANGLADESH	BRUNEI	1
BANGLADESH	EGYPT	1
BANGLADESH	SAUDI ARABIA	1
BELGIUM	BOLIVIA	1
BRAZIL	ARGENTINA	1
BRAZIL	CHILE	1
BRAZIL	ECUADOR	2
BRAZIL	MEXICO	1
BRAZIL	PANAMA	1
CANADA	BRAZIL	1
CANADA	FINLAND	1
CANADA	FRANCE	1
CANADA	SPAIN	1
CANADA	SWEDEN	1
CANADA	SWITZERLAND	1
CHILE	PANAMA	1
CHINA	AUSTRALIA	7
CHINA	BANGLADESH	1
CHINA	BELGIUM	3
CHINA	CANADA	7
CHINA	COLOMBIA	1
CHINA	CZECH REPUBLIC	2
CHINA	DENMARK	3
CHINA	EGYPT	1
CHINA	FINLAND	1
CHINA	GERMANY	2

CHINA	INDIA	3
CHINA	INDONESIA	2
CHINA	ITALY	2
CHINA	KOREA	3
CHINA	MALAYSIA	3
CHINA	NETHERLANDS	1
CHINA	NEW ZEALAND	1
CHINA	NORWAY	1
CHINA	PAKISTAN	1
CHINA	RUSSIA	1
CHINA	SAUDI ARABIA	2
CHINA	SPAIN	2
CHINA	SWEDEN	1
CHINA	SWITZERLAND	2
CHINA	THAILAND	2
CHINA	UNITED KINGDOM	11
CHINA	USA	14
CZECH REPUBLIC	SERBIA	1
ECUADOR	MEXICO	2
EGYPT	BRUNEI	1
ESTONIA	SLOVENIA	1
FIJI	BELIZE	1
FINLAND	AUSTRIA	1
FINLAND	SOUTH AFRICA	3
FINLAND	SWEDEN	1
FINLAND	SWITZERLAND	1
FINLAND	TANZANIA	1
FRANCE	BELGIUM	1
FRANCE	ECUADOR	1
FRANCE	KUWAIT	1
FRANCE	MEXICO	1
FRANCE	SWITZERLAND	2
FRANCE	TUNISIA	1
FRANCE	VIETNAM	3
GERMANY	CANADA	1
GERMANY	COSTA RICA	1
GERMANY	ESTONIA	1
GERMANY	FINLAND	4
GERMANY	FRANCE	1
GERMANY	INDIA	1
GERMANY	IRELAND	2
GERMANY	ITALY	5
GERMANY	NETHERLANDS	4
GERMANY	NEW ZEALAND	1

GERMANY	NIGERIA	2
GERMANY	NORWAY	6
GERMANY	ROMANIA	1
GERMANY	SOUTH AFRICA	2
GERMANY	SPAIN	1
GERMANY	SWEDEN	2
GERMANY	SWITZERLAND	2
GERMANY	UNITED KINGDOM	3
INDIA	AUSTRALIA	1
INDIA	BANGLADESH	2
INDIA	BRUNEI	1
INDIA	CZECH REPUBLIC	1
INDIA	EGYPT	1
INDIA	ETHIOPIA	1
INDIA	FINLAND	1
INDIA	JAPAN	1
INDIA	KOREA	2
INDIA	MALAYSIA	2
INDIA	MEXICO	2
INDIA	NETHERLANDS	1
INDIA	SAUDI ARABIA	3
INDIA	SOUTH AFRICA	1
INDIA	SWEDEN	1
INDIA	VIETNAM	1
INDIA	ZAMBIA	1
INDONESIA	MALAYSIA	2
INDONESIA	SAUDI ARABIA	1
IRAN	BELGIUM	1
IRAN	FRANCE	1
IRAN	LITHUANIA	2
IRAN	POLAND	3
ITALY	AUSTRALIA	1
ITALY	BRAZIL	1
ITALY	COSTA RICA	1
ITALY	FINLAND	1
ITALY	IRAN	1
ITALY	LITHUANIA	1
ITALY	POLAND	1
ITALY	RUSSIA	1
ITALY	SAUDI ARABIA	1
ITALY	SWEDEN	1
ITALY	SWITZERLAND	1
JAPAN	BANGLADESH	1
JAPAN	INDONESIA	1

JAPAN	MONGOLIA	1
JAPAN	RUSSIA	1
JAPAN	SOUTH AFRICA	1
JAPAN	THAILAND	2
KOREA	BANGLADESH	1
KOREA	EGYPT	1
KOREA	INDONESIA	1
KOREA	MALAYSIA	1
KOREA	SAUDI ARABIA	2
KOREA	ZAMBIA	1
KUWAIT	U ARAB EMIRATES	1
MALAYSIA	BANGLADESH	1
MALAYSIA	BRUNEI	1
MALAYSIA	EGYPT	1
MALAYSIA	SAUDI ARABIA	2
MALAYSIA	SOUTH AFRICA	1
MALAYSIA	THAILAND	1
MEXICO	ETHIOPIA	1
NETHERLANDS	ETHIOPIA	1
NETHERLANDS	IRELAND	2
NETHERLANDS	NEW ZEALAND	1
NETHERLANDS	UKRAINE	1
NORWAY	CZECH REPUBLIC	1
NORWAY	DENMARK	1
NORWAY	NIGERIA	1
PAKISTAN	HUNGARY	1
PAKISTAN	SAUDI ARABIA	1
POLAND	LITHUANIA	2
PORTUGAL	AUSTRIA	1
PORTUGAL	BRAZIL	1
PORTUGAL	CZECH REPUBLIC	1
RUSSIA	NORWAY	2
SAUDI ARABIA	BRUNEI	1
SAUDI ARABIA	EGYPT	1
SAUDI ARABIA	ZAMBIA	1
SOUTH AFRICA	GHANA	1
SPAIN	ARGENTINA	1
SPAIN	BELGIUM	3
SPAIN	BELIZE	1
SPAIN	BRAZIL	2
SPAIN	CHILE	2
SPAIN	CZECH REPUBLIC	1
SPAIN	DENMARK	1
SPAIN	ECUADOR	1

SPAIN	FIJI	1
SPAIN	FINLAND	1
SPAIN	FRANCE	3
SPAIN	MEXICO	2
SPAIN	NORWAY	1
SPAIN	PANAMA	1
SPAIN	PORTUGAL	1
SPAIN	SAUDI ARABIA	2
SPAIN	SWITZERLAND	1
SPAIN	URUGUAY	1
SWEDEN	ICELAND	1
SWEDEN	IRELAND	1
SWEDEN	SAUDI ARABIA	1
THAILAND	NEPAL	2
TURKEY	CZECH REPUBLIC	1
TURKEY	SERBIA	1
UNITED KINGDOM	AUSTRALIA	1
UNITED KINGDOM	BANGLADESH	1
UNITED KINGDOM	BELGIUM	1
UNITED KINGDOM	COLOMBIA	1
UNITED KINGDOM	CZECH REPUBLIC	1
UNITED KINGDOM	DENMARK	4
UNITED KINGDOM	FRANCE	5
UNITED KINGDOM	INDIA	1
UNITED KINGDOM	INDONESIA	1
UNITED KINGDOM	IRAN	5
UNITED KINGDOM	IRELAND	1
UNITED KINGDOM	KOREA	1
UNITED KINGDOM	LITHUANIA	1
UNITED KINGDOM	NETHERLANDS	2
UNITED KINGDOM	NEW ZEALAND	1
UNITED KINGDOM	NORWAY	3
UNITED KINGDOM	POLAND	2
UNITED KINGDOM	ROMANIA	1
UNITED KINGDOM	RUSSIA	1
UNITED KINGDOM	SERBIA	1
UNITED KINGDOM	SPAIN	1
UNITED KINGDOM	SWEDEN	1
UNITED KINGDOM	SWITZERLAND	1
UNITED KINGDOM	TURKEY	1
USA	ARGENTINA	1
USA	AUSTRALIA	1
USA	BANGLADESH	2
USA	BRAZIL	1

USA	CANADA	5
USA	COLOMBIA	1
USA	CROATIA	2
USA	CZECH REPUBLIC	2
USA	DENMARK	2
USA	FRANCE	3
USA	GERMANY	2
USA	INDIA	2
USA	ITALY	1
USA	JAPAN	1
USA	KOREA	2
USA	KUWAIT	1
USA	PAKISTAN	1
USA	RUSSIA	1
USA	SAUDI ARABIA	1
USA	SERBIA	2
USA	SPAIN	3
USA	SWEDEN	1
USA	TURKEY	1
USA	UNITED KINGDOM	5
VIETNAM	MEXICO	1

Table S2. Countries involved in microplastics-related research and the volume of their publications, 2018-2022

**From**	**To**	**Frequency**
ARGENTINA	URUGUAY	2
AUSTRALIA	BANGLADESH	1
AUSTRALIA	BRUNEI	1
AUSTRALIA	EGYPT	1
AUSTRALIA	GHANA	1
AUSTRALIA	IRAN	1
AUSTRALIA	LITHUANIA	1
AUSTRALIA	MALAYSIA	1
AUSTRALIA	NORWAY	1
AUSTRALIA	PAKISTAN	1
AUSTRALIA	POLAND	1
AUSTRALIA	RUSSIA	2
AUSTRALIA	SAUDI ARABIA	1
AUSTRALIA	THAILAND	1
AUSTRALIA	VIETNAM	1
BANGLADESH	BRUNEI	1
BANGLADESH	EGYPT	1
BANGLADESH	SAUDI ARABIA	1
BELGIUM	BOLIVIA	1

BRAZIL	ARGENTINA	1
BRAZIL	CHILE	1
BRAZIL	ECUADOR	2
BRAZIL	MEXICO	1
BRAZIL	PANAMA	1
CANADA	BRAZIL	1
CANADA	FINLAND	1
CANADA	FRANCE	1
CANADA	SPAIN	1
CANADA	SWEDEN	1
CANADA	SWITZERLAND	1
CHILE	PANAMA	1
CHINA	AUSTRALIA	7
CHINA	BANGLADESH	1
CHINA	BELGIUM	3
CHINA	CANADA	7
CHINA	COLOMBIA	1
CHINA	CZECH REPUBLIC	2
CHINA	DENMARK	3
CHINA	EGYPT	1
CHINA	FINLAND	1
CHINA	GERMANY	2
CHINA	INDIA	3
CHINA	INDONESIA	2
CHINA	ITALY	2
CHINA	KOREA	3
CHINA	MALAYSIA	3
CHINA	NETHERLANDS	1
CHINA	NEW ZEALAND	1
CHINA	NORWAY	1
CHINA	PAKISTAN	1
CHINA	RUSSIA	1
CHINA	SAUDI ARABIA	2
CHINA	SPAIN	2
CHINA	SWEDEN	1
CHINA	SWITZERLAND	2
CHINA	THAILAND	2
CHINA	UNITED KINGDOM	11
CHINA	USA	14
CZECH REPUBLIC	SERBIA	1
ECUADOR	MEXICO	2
EGYPT	BRUNEI	1
ESTONIA	SLOVENIA	1
FIJI	BELIZE	1

FINLAND	AUSTRIA	1
FINLAND	SOUTH AFRICA	3
FINLAND	SWEDEN	1
FINLAND	SWITZERLAND	1
FINLAND	TANZANIA	1
FRANCE	BELGIUM	1
FRANCE	ECUADOR	1
FRANCE	KUWAIT	1
FRANCE	MEXICO	1
FRANCE	SWITZERLAND	2
FRANCE	TUNISIA	1
FRANCE	VIETNAM	3
GERMANY	CANADA	1
GERMANY	COSTA RICA	1
GERMANY	ESTONIA	1
GERMANY	FINLAND	4
GERMANY	FRANCE	1
GERMANY	INDIA	1
GERMANY	IRELAND	2
GERMANY	ITALY	5
GERMANY	NETHERLANDS	4
GERMANY	NEW ZEALAND	1
GERMANY	NIGERIA	2
GERMANY	NORWAY	6
GERMANY	ROMANIA	1
GERMANY	SOUTH AFRICA	2
GERMANY	SPAIN	1
GERMANY	SWEDEN	2
GERMANY	SWITZERLAND	2
GERMANY	UNITED KINGDOM	3
INDIA	AUSTRALIA	1
INDIA	BANGLADESH	2
INDIA	BRUNEI	1
INDIA	CZECH REPUBLIC	1
INDIA	EGYPT	1
INDIA	ETHIOPIA	1
INDIA	FINLAND	1
INDIA	JAPAN	1
INDIA	KOREA	2
INDIA	MALAYSIA	2
INDIA	MEXICO	2
INDIA	NETHERLANDS	1
INDIA	SAUDI ARABIA	3
INDIA	SOUTH AFRICA	1

INDIA	SWEDEN	1
INDIA	VIETNAM	1
INDIA	ZAMBIA	1
INDONESIA	MALAYSIA	2
INDONESIA	SAUDI ARABIA	1
IRAN	BELGIUM	1
IRAN	FRANCE	1
IRAN	LITHUANIA	2
IRAN	POLAND	3
ITALY	AUSTRALIA	1
ITALY	BRAZIL	1
ITALY	COSTA RICA	1
ITALY	FINLAND	1
ITALY	IRAN	1
ITALY	LITHUANIA	1
ITALY	POLAND	1
ITALY	RUSSIA	1
ITALY	SAUDI ARABIA	1
ITALY	SWEDEN	1
ITALY	SWITZERLAND	1
JAPAN	BANGLADESH	1
JAPAN	INDONESIA	1
JAPAN	MONGOLIA	1
JAPAN	RUSSIA	1
JAPAN	SOUTH AFRICA	1
JAPAN	THAILAND	2
KOREA	BANGLADESH	1
KOREA	EGYPT	1
KOREA	INDONESIA	1
KOREA	MALAYSIA	1
KOREA	SAUDI ARABIA	2
KOREA	ZAMBIA	1
KUWAIT	U ARAB EMIRATES	1
MALAYSIA	BANGLADESH	1
MALAYSIA	BRUNEI	1
MALAYSIA	EGYPT	1
MALAYSIA	SAUDI ARABIA	2
MALAYSIA	SOUTH AFRICA	1
MALAYSIA	THAILAND	1
MEXICO	ETHIOPIA	1
NETHERLANDS	ETHIOPIA	1
NETHERLANDS	IRELAND	2
NETHERLANDS	NEW ZEALAND	1
NETHERLANDS	UKRAINE	1

NORWAY	CZECH REPUBLIC	1
NORWAY	DENMARK	1
NORWAY	NIGERIA	1
PAKISTAN	HUNGARY	1
PAKISTAN	SAUDI ARABIA	1
POLAND	LITHUANIA	2
PORTUGAL	AUSTRIA	1
PORTUGAL	BRAZIL	1
PORTUGAL	CZECH REPUBLIC	1
RUSSIA	NORWAY	2
SAUDI ARABIA	BRUNEI	1
SAUDI ARABIA	EGYPT	1
SAUDI ARABIA	ZAMBIA	1
SOUTH AFRICA	GHANA	1
SPAIN	ARGENTINA	1
SPAIN	BELGIUM	3
SPAIN	BELIZE	1
SPAIN	BRAZIL	2
SPAIN	CHILE	2
SPAIN	CZECH REPUBLIC	1
SPAIN	DENMARK	1
SPAIN	ECUADOR	1
SPAIN	FIJI	1
SPAIN	FINLAND	1
SPAIN	FRANCE	3
SPAIN	MEXICO	2
SPAIN	NORWAY	1
SPAIN	PANAMA	1
SPAIN	PORTUGAL	1
SPAIN	SAUDI ARABIA	2
SPAIN	SWITZERLAND	1
SPAIN	URUGUAY	1
SWEDEN	ICELAND	1
SWEDEN	IRELAND	1
SWEDEN	SAUDI ARABIA	1
THAILAND	NEPAL	2
TURKEY	CZECH REPUBLIC	1
TURKEY	SERBIA	1
UNITED KINGDOM	AUSTRALIA	1
UNITED KINGDOM	BANGLADESH	1
UNITED KINGDOM	BELGIUM	1
UNITED KINGDOM	COLOMBIA	1
UNITED KINGDOM	CZECH REPUBLIC	1
UNITED KINGDOM	DENMARK	4

UNITED KINGDOM	FRANCE	5
UNITED KINGDOM	INDIA	1
UNITED KINGDOM	INDONESIA	1
UNITED KINGDOM	IRAN	5
UNITED KINGDOM	IRELAND	1
UNITED KINGDOM	KOREA	1
UNITED KINGDOM	LITHUANIA	1
UNITED KINGDOM	NETHERLANDS	2
UNITED KINGDOM	NEW ZEALAND	1
UNITED KINGDOM	NORWAY	3
UNITED KINGDOM	POLAND	2
UNITED KINGDOM	ROMANIA	1
UNITED KINGDOM	RUSSIA	1
UNITED KINGDOM	SERBIA	1
UNITED KINGDOM	SPAIN	1
UNITED KINGDOM	SWEDEN	1
UNITED KINGDOM	SWITZERLAND	1
UNITED KINGDOM	TURKEY	1
USA	ARGENTINA	1
USA	AUSTRALIA	1
USA	BANGLADESH	2
USA	BRAZIL	1
USA	CANADA	5
USA	COLOMBIA	1
USA	CROATIA	2
USA	CZECH REPUBLIC	2
USA	DENMARK	2
USA	FRANCE	3
USA	GERMANY	2
USA	INDIA	2
USA	ITALY	1
USA	JAPAN	1
USA	KOREA	2
USA	KUWAIT	1
USA	PAKISTAN	1
USA	RUSSIA	1
USA	SAUDI ARABIA	1
USA	SERBIA	2
USA	SPAIN	3
USA	SWEDEN	1
USA	TURKEY	1
USA	UNITED KINGDOM	5
VIETNAM	MEXICO	1
ARGENTINA	URUGUAY	2

AUSTRALIA	BANGLADESH	1
AUSTRALIA	BRUNEI	1
AUSTRALIA	EGYPT	1
AUSTRALIA	GHANA	1
AUSTRALIA	IRAN	1
AUSTRALIA	LITHUANIA	1
AUSTRALIA	MALAYSIA	1
AUSTRALIA	NORWAY	1
AUSTRALIA	PAKISTAN	1
AUSTRALIA	POLAND	1
AUSTRALIA	RUSSIA	2
AUSTRALIA	SAUDI ARABIA	1
AUSTRALIA	THAILAND	1
AUSTRALIA	VIETNAM	1
BANGLADESH	BRUNEI	1
BANGLADESH	EGYPT	1
BANGLADESH	SAUDI ARABIA	1
BELGIUM	BOLIVIA	1
BRAZIL	ARGENTINA	1
BRAZIL	CHILE	1
BRAZIL	ECUADOR	2
BRAZIL	MEXICO	1
BRAZIL	PANAMA	1
CANADA	BRAZIL	1
CANADA	FINLAND	1
CANADA	FRANCE	1
CANADA	SPAIN	1
CANADA	SWEDEN	1
CANADA	SWITZERLAND	1
CHILE	PANAMA	1
CHINA	AUSTRALIA	7
CHINA	BANGLADESH	1
CHINA	BELGIUM	3
CHINA	CANADA	7
CHINA	COLOMBIA	1
CHINA	CZECH REPUBLIC	2
CHINA	DENMARK	3
CHINA	EGYPT	1
CHINA	FINLAND	1
CHINA	GERMANY	2
CHINA	INDIA	3
CHINA	INDONESIA	2
CHINA	ITALY	2
CHINA	KOREA	3

CHINA	MALAYSIA	3
CHINA	NETHERLANDS	1
CHINA	NEW ZEALAND	1
CHINA	NORWAY	1
CHINA	PAKISTAN	1
CHINA	RUSSIA	1
CHINA	SAUDI ARABIA	2
CHINA	SPAIN	2
CHINA	SWEDEN	1
CHINA	SWITZERLAND	2
CHINA	THAILAND	2
CHINA	UNITED KINGDOM	11
CHINA	USA	14
CZECH REPUBLIC	SERBIA	1
ECUADOR	MEXICO	2
EGYPT	BRUNEI	1
ESTONIA	SLOVENIA	1
FIJI	BELIZE	1
FINLAND	AUSTRIA	1
FINLAND	SOUTH AFRICA	3
FINLAND	SWEDEN	1
FINLAND	SWITZERLAND	1
FINLAND	TANZANIA	1
FRANCE	BELGIUM	1
FRANCE	ECUADOR	1
FRANCE	KUWAIT	1
FRANCE	MEXICO	1
FRANCE	SWITZERLAND	2
FRANCE	TUNISIA	1
FRANCE	VIETNAM	3
GERMANY	CANADA	1
GERMANY	COSTA RICA	1
GERMANY	ESTONIA	1
GERMANY	FINLAND	4
GERMANY	FRANCE	1
GERMANY	INDIA	1
GERMANY	IRELAND	2
GERMANY	ITALY	5
GERMANY	NETHERLANDS	4
GERMANY	NEW ZEALAND	1
GERMANY	NIGERIA	2
GERMANY	NORWAY	6
GERMANY	ROMANIA	1
GERMANY	SOUTH AFRICA	2

GERMANY	SPAIN	1
GERMANY	SWEDEN	2
GERMANY	SWITZERLAND	2
GERMANY	UNITED KINGDOM	3
INDIA	AUSTRALIA	1
INDIA	BANGLADESH	2
INDIA	BRUNEI	1
INDIA	CZECH REPUBLIC	1
INDIA	EGYPT	1
INDIA	ETHIOPIA	1
INDIA	FINLAND	1
INDIA	JAPAN	1
INDIA	KOREA	2
INDIA	MALAYSIA	2
INDIA	MEXICO	2
INDIA	NETHERLANDS	1
INDIA	SAUDI ARABIA	3
INDIA	SOUTH AFRICA	1
INDIA	SWEDEN	1
INDIA	VIETNAM	1
INDIA	ZAMBIA	1
INDONESIA	MALAYSIA	2
INDONESIA	SAUDI ARABIA	1
IRAN	BELGIUM	1
IRAN	FRANCE	1
IRAN	LITHUANIA	2
IRAN	POLAND	3
ITALY	AUSTRALIA	1
ITALY	BRAZIL	1
ITALY	COSTA RICA	1
ITALY	FINLAND	1
ITALY	IRAN	1
ITALY	LITHUANIA	1
ITALY	POLAND	1
ITALY	RUSSIA	1
ITALY	SAUDI ARABIA	1
ITALY	SWEDEN	1
ITALY	SWITZERLAND	1
JAPAN	BANGLADESH	1
JAPAN	INDONESIA	1
JAPAN	MONGOLIA	1
JAPAN	RUSSIA	1
JAPAN	SOUTH AFRICA	1
JAPAN	THAILAND	2

KOREA	BANGLADESH	1
KOREA	EGYPT	1
KOREA	INDONESIA	1
KOREA	MALAYSIA	1
KOREA	SAUDI ARABIA	2
KOREA	ZAMBIA	1
KUWAIT	U ARAB EMIRATES	1
MALAYSIA	BANGLADESH	1
MALAYSIA	BRUNEI	1
MALAYSIA	EGYPT	1
MALAYSIA	SAUDI ARABIA	2
MALAYSIA	SOUTH AFRICA	1
MALAYSIA	THAILAND	1
MEXICO	ETHIOPIA	1
NETHERLANDS	ETHIOPIA	1
NETHERLANDS	IRELAND	2
NETHERLANDS	NEW ZEALAND	1
NETHERLANDS	UKRAINE	1
NORWAY	CZECH REPUBLIC	1
NORWAY	DENMARK	1
NORWAY	NIGERIA	1
PAKISTAN	HUNGARY	1
PAKISTAN	SAUDI ARABIA	1
POLAND	LITHUANIA	2
PORTUGAL	AUSTRIA	1
PORTUGAL	BRAZIL	1
PORTUGAL	CZECH REPUBLIC	1
RUSSIA	NORWAY	2
SAUDI ARABIA	BRUNEI	1
SAUDI ARABIA	EGYPT	1
SAUDI ARABIA	ZAMBIA	1
SOUTH AFRICA	GHANA	1
SPAIN	ARGENTINA	1
SPAIN	BELGIUM	3
SPAIN	BELIZE	1
SPAIN	BRAZIL	2
SPAIN	CHILE	2
SPAIN	CZECH REPUBLIC	1
SPAIN	DENMARK	1
SPAIN	ECUADOR	1
SPAIN	FIJI	1
SPAIN	FINLAND	1
SPAIN	FRANCE	3
SPAIN	MEXICO	2

SPAIN	NORWAY	1
SPAIN	PANAMA	1
SPAIN	PORTUGAL	1
SPAIN	SAUDI ARABIA	2
SPAIN	SWITZERLAND	1
SPAIN	URUGUAY	1
SWEDEN	ICELAND	1
SWEDEN	IRELAND	1
SWEDEN	SAUDI ARABIA	1
THAILAND	NEPAL	2
TURKEY	CZECH REPUBLIC	1
TURKEY	SERBIA	1
UNITED KINGDOM	AUSTRALIA	1
UNITED KINGDOM	BANGLADESH	1
UNITED KINGDOM	BELGIUM	1
UNITED KINGDOM	COLOMBIA	1
UNITED KINGDOM	CZECH REPUBLIC	1
UNITED KINGDOM	DENMARK	4
UNITED KINGDOM	FRANCE	5
UNITED KINGDOM	INDIA	1
UNITED KINGDOM	INDONESIA	1
UNITED KINGDOM	IRAN	5
UNITED KINGDOM	IRELAND	1
UNITED KINGDOM	KOREA	1
UNITED KINGDOM	LITHUANIA	1
UNITED KINGDOM	NETHERLANDS	2
UNITED KINGDOM	NEW ZEALAND	1
UNITED KINGDOM	NORWAY	3
UNITED KINGDOM	POLAND	2
UNITED KINGDOM	ROMANIA	1
UNITED KINGDOM	RUSSIA	1
UNITED KINGDOM	SERBIA	1
UNITED KINGDOM	SPAIN	1
UNITED KINGDOM	SWEDEN	1
UNITED KINGDOM	SWITZERLAND	1
UNITED KINGDOM	TURKEY	1
USA	ARGENTINA	1
USA	AUSTRALIA	1
USA	BANGLADESH	2
USA	BRAZIL	1
USA	CANADA	5
USA	COLOMBIA	1
USA	CROATIA	2
USA	CZECH REPUBLIC	2

USA	DENMARK	2
USA	FRANCE	3
USA	GERMANY	2
USA	INDIA	2
USA	ITALY	1
USA	JAPAN	1
USA	KOREA	2
USA	KUWAIT	1
USA	PAKISTAN	1
USA	RUSSIA	1
USA	SAUDI ARABIA	1
USA	SERBIA	2
USA	SPAIN	3
USA	SWEDEN	1
USA	TURKEY	1
USA	UNITED KINGDOM	5
VIETNAM	MEXICO	1
